# Vitamin D status of healthy adolescents from two states in Malaysia

**DOI:** 10.1186/1687-9856-2015-S1-P62

**Published:** 2015-04-28

**Authors:** Suhaimi Hussain, Maged Elnajeh, Muhammad Yazid Jalaludin, Fatimah Harun

**Affiliations:** 1 Paediatric Department School of Medical Sciences Hospital University Science Malaysia, Kota Bharu, Kelantan, Malaysia; 2 Department of Pediatrics, Faculty of Medicine, University of Malaya, Kuala Lumpur, Malaysia

## 

Hypovitaminosis D is a widespread disorder across all age groups in developing countries. The prevalence of hypovitaminosis D varies from 30-90% depending on the cut off level used to define hypovitaminosis D. In Malaysia, Khor et. al found 35.3% of 402 primary school children aged 7-12 years to have 25(OH)D level < 37.5nmol/L and 37.1% have the level between 37.5-50nmol/L. If a broader definition of hypovitaminosis < 50nmol/L is used then, the prevalence was as high as 74.6%. The risk factors associated with hypovitaminosis D in developing countries are the same as in western countries. The most consistently reported risk factors are female gender, increased skin pigmentation, seasons/latitudes, obesity, concealing clothing style and vulnerable groups (neonates, preschool, elderly).

A total of 469 adolescents (107 PJ, 362 KB) participated in the study. The mean age was 15.6+/-1.4 years. Female gender contributed about 61.0% compared to male gender, 39.1%. As for the race distribution, the proportion of Malay was 79.3%, Chinese 17.7% and Indian 3.0%. Teenagers from KB with family income < RM 1000 was higher (37.8% cf 10.3%; P <0.001). Adolescents from PJ was taller (160.6cm cf 156.3cm; P = 0.02). The mean BMI was 21.0+/-4.4 kgm^2^. The mean 25(OH)D was19.9+/-8.1, in which PJ had a higher level(21.0 cf 19.6) but the mean differences was not statistically significant. More than half (58%) of adolescents had 25(OH)D < 50nmol/L. The proportion of subjects with 25(OH)D < 50nmol/L was 60.2% in KB and 50.4% in PJ. With regard to the degree of 25(OH)D level, 52% had a level between 25.0-50.0nmol/L, 6% had a level between 12.5-25.0nmol/L. None had a level < 12.5nmol/L.

Chinese had the highest mean of 25(OH)D(23.5+/-8.0; P <0.001) compared to the other 2 races. From multiple logistic regression, significant variables were age (0.84;95%CI :0.72,0.98), gender(boy)(0.13;95%CI;0.08,0.19) and race(Chinese)(0.30;05%C1;0.17,0.53). This study highlights a high prevalence of hypovitaminosis D among adolescents especially in younger adolescents, female and darker skin.

